# Fast and Efficient Transfection of Mouse Embryonic Stem Cells Using Non-Viral Reagents

**DOI:** 10.1007/s12015-016-9673-5

**Published:** 2016-06-30

**Authors:** Christoffer Tamm, Sandeep Kadekar, Sara Pijuan-Galitó, Cecilia Annerén

**Affiliations:** 1 Department of Medical Biochemistry and Microbiology, Uppsala University, Box 582, SE-751 23 Uppsala, Sweden; 2 GE Healthcare BioSciences AB, Björkgatan 30, Uppsala, SE-751 84 Sweden

**Keywords:** Transfection, Mouse, Embryonic stem cells, ES cells, siRNA, DNA, Plasmid

## Abstract

Reliable and efficient DNA and RNA transfection methods are required when studying the role of individual genes in mouse pluripotent stem cells. However, these cells usually grow in tight clusters and are therefore more difficult to transfect than many other cell lines. We have found that transfection is especially challenging when mouse embryonic stem (mES) cells are cultured in the newly described 2i medium, which is based on two chemical inhibitors of differentiation pathways. In the present study we have performed a side-by-side comparison of commercially available, non-viral transfection reagents with regard to their ability to deliver plasmid DNA and siRNA into adherent and/or trypsinized mES cells cultured in 2i medium, assessing transfection rates, plasmid gene expression, siRNA mediated knockdown of Oct4 and viability. Finally, we present a fast and efficient method for transfection of trypsinized mES cells using the liposomal-based Lipofectamine 2000. With only a five-minute long transfection time we obtained at least 85 % transfected cells with 80 % maintained viability. Moreover, this protocol saves up to a day of experimental time since the cells are in suspension at the time of transfection, which allows for immediately re-plating into the appropriate format. This fast, simplified and highly efficient transfection method will be valuable for both basic research and high-throughput applications.

**Electronic supplementary material:**

The online version of this article (doi:10.1007/s12015-016-9673-5) contains supplementary material, which is available to authorized users.

## Introduction

For the last decades mouse embryonic stem (mES) cells have been considered to be one of the most interesting model systems for basic cellular and molecular studies and developmental research. The ability to efficiently transfer DNA or small interfering RNA (siRNA) is one of the key technologies used for studying the role of single genes in these cells. However, mES cells grow in tight clusters and are therefore quite inefficiently transfected using chemical reagents. In addition, we have found that when mES cells are cultured in the serum-free 2i medium (containing two inhibitors, against the mitogen-activated protein kinase (MAPK)/extracellular-signal-regulated kinase (ERK) kinase (MEK) and the glycogen synthase kinase 3 (GSK3), respectively [1]), chemical transfection is even more challenging as compared to when cells are cultured in standard culture media [2]. Replacement of serum by targeted inhibition of relevant signaling pathways reduces cellular heterogeneity as well as transcriptome and epigenome differences in mES cell cultures [3 , 4 ]. In 2i medium PD0325901 inhibits the autocrine activation of the ERK1/2 pathway by fibroblast growth factor-4 (FGF4), shown to be instrumental for ES cell differentiation [5 ]. The GSK3 inhibition impairs Tcf3 activity, which represses the core pluripotency network in mES cells [6 ]. The cell cultures exhibit a more dense and uniform morphology, with negligible signs of spontaneous differentiation as compared to cells cultured in serum-containing media [2].

There are many vastly different transfection techniques available [7 ]. Virus-mediated transfections, or so-called biological transfection methods, are suitable for stable transfections due to the integration of transfected DNA into the host cell genome. However, although viral methods are usually highly efficient they suffer from limitations such as time-consuming production of vectors, mutagenesis, cytotoxicity, and DNA size restriction [8 ]. Physical transfection methods are the means of diverse physical tools to deliver nucleic acids into host cells. Despite being effective, these methods are both complicated and time-consuming (e.g. micro injections) or may cause massive cell death (e.g. electroporation and nucleofection). En masse they also require expensive in-house equipment. Instead, much effort has been put into the development of chemical methods, such as calcium phosphates, cationic lipids, cationic polymers, dendrimers, polyethylenimine, peptides and nanoparticle-based compounds [7 ]. Although generally less efficient in delivering DNA, these non-viral vectors have higher genetic material carrying capacity, are easier to produce and cause low cytotoxicity. However, many challenges remain, such as increasing transfection efficacy while maintaining or reducing cytotoxicity, as well as improving penetration of cells that are difficult to transfect.

Here we present a side-by-side comparison of commercially available, non-viral transfection reagents for plasmid DNA or siRNA delivery using the manufacturers’ instructions. For DNA and RNA delivery we selected some of the most commonly used reagents on the market, both liposomal and non-liposomal polymer based. In addition we have also used a positively charged polymer embedded into a porous carrier nanoparticle. One of the unique challenges when transfecting mES cells is that these cells often grow in dense multilayered colonies, leading to lower transfection rates in the cells that are positioned in the middle of the colonies. Hence, in the present study we have transfected both adherent colonies as well as cells dispersed into single cell suspensions. We here present an optimized, rapid, single-cell suspension transfection protocol, which results in unparalleled efficacy while maintaining low post-transfection toxicity.

## Material and Methods

### Embryonic Stem Cell Culturing

Feeder-independent E14 and R1 mES cells were cultured in 2i medium [1], a serum-free N2B27 medium supplemented with MEK inhibitor PD0325901 (1 μM) and GSK3 inhibitor CHIR99021 (3 μM) (both from Selleckchem), and 1000 U/ml LIF (Millipore). TrypLE™ Express (Life Technologies) was used to passage the cells. To ensure good adherence 2 % ES-qualified FBS was supplemented to the 2i media when seeding the cells, as previously described [2].

### Transfections

Transfections with Lipofectamine® 2000 (herein named L2K), Lipofectamine® 3000 (L3K, Life Technologies), TurboFect™ (GE Healthcare Dharmacon), FuGENE® HD, ViaFect™ (Promega), TransIT-2020®, TransIT-X2®, TransIT-TKO®, TransIT-siQUEST® (Mirus Bio), Xfect™ mESC (Clontech), X-tremeGENE™ HP, X-tremeGENE™ 9 (Roche), Nanofectin, Nanofectamin (PAA, now part of GE Healthcare), JetPrime™ (Polyplus transfection) were performed according to the manufacturer’s instructions within the recommended reagent/DNA or reagent/siRNA ratio range. For adherent plasmid-DNA transfections cells were plated at constant density 2 x 10^5^ cells/well in 12 well-plates one day prior to transfection. For the single cell suspension; 5 x 10^5^ cells/well were transfected and plated in 12 well-plates. The pmaxGFP (AMAXA Biosystems), pCMV β-galactosidase, or pCAGGs heparanase vectors were introduced at a final concentration of 1.5 μg DNA/well. For the adherent siRNA transfections; cells were plated at a density 5 x 10^4^ cells/well in 24 well plates one day prior to transfection with either scrambled siRNA or siRNA against Oct4 at a final concentration of 50 or 100 nM/well. For the siRNA single cell suspension transfections; cells were transfected and plated at a density 5 x 10^4^ cells/well in a 24 well plates. (sense 5′-AGGUGUUCAGCCAGACCACTT-3′, antisense 5′-GUGGUCUGGCUGAACACCUTT-3′).

### Flow Cytometry

24 h post-transfection, cells were washed once with PBS and then trypsinized using TrypLE™ Express for 5 min at 37 °C. Cell suspensions were analysed in a Becton Dickinson FACSCalibur flow cytometer for FL1 (GFP) fluorescence to determine the percentage of reporter expressing cells. Background fluorescence and autofluorescence were determined using non-transfected cells as control. Analysis was carried out in the freeware Flowing Software (http://www.flowingsoftware.com).

### Western Blot

Cells were harvested in lysis buffer as previously described [9 ]. Total protein concentration was measured using BCA Protein Assay kit (Pierce). Samples were run on 10 % SDS PAGE gels, and subsequently transferred to an Immobilon-FL membrane (Millipore). The membranes were blotted with anti-Oct3/4 (POUF5F1, cl. 7F9.2 (Millipore) and anti-β actin ab8229 (Abcam). The membranes were then incubated with Alexa Fluor anti-rabbit 680 and IRDye 800CW anti-mouse. Immunosignals were imaged using an Odyssey fluorescent imaging scanner (LI-COR Biosciences).

### Β-Galactosidase Activity Assay

Twenty-four hours post-transfection cells were harvested in lysis buffer and mixed with ONPG buffer (60 mM Na_2_HPO_4_, 40 mM NaH_2_PO_4_, 10 mM KCl, 1 mM MgCl_2_, 50 mM 2-mercaptoethanol, 1.2 mM ONPG) in a microtiter plate and incubated at 37 °C for 30 min. Absorbance was read at 420 nm in a microplate photometer reader (with Multiscan® PLUS, Labsystems).

### qPCR

Twenty four and 48 h post-transfection total RNA was extracted and purified with the GenElute™ mammalian total RNA miniprep kit (Sigma-Aldrich) according to the manufacturer’s instructions. First-strand cDNA was produced according to the manufacturer’s protocol with iScript™ cDNA synthesis kit (Bio-Rad) using 1 μg RNA. Quantitative real-time PCR was performed according to the manufacturer’s instructions using the SsoFast™ EvaGreen™ supermix on the Miniopticon™ Real-Time PCR Detection System (Bio-Rad). The average C(t) value for each gene was normalized against 18S and the comparative *C*_*t*_ value (fold change) was calculated using 2^−ΔΔ*C*(*t*)^. Primers used were: 18S (forward: AGTCCCTGCCCTTTGTACACA, reverse: GATCCGAGGGCCTCACTAAAC).

Oct4 (forward:GATGCTGTGAGCCCAAG-GCAAG, reverse: GGCTCCTGATCAACAGCATCAC),

Dab2 (forward: TGAAGCAGACAGCCAGAACA, reverse: CAACAGACAAGGATTTGATAGGG).

## Results

### Efficiency of siRNA Delivery

**Fig. 1:**
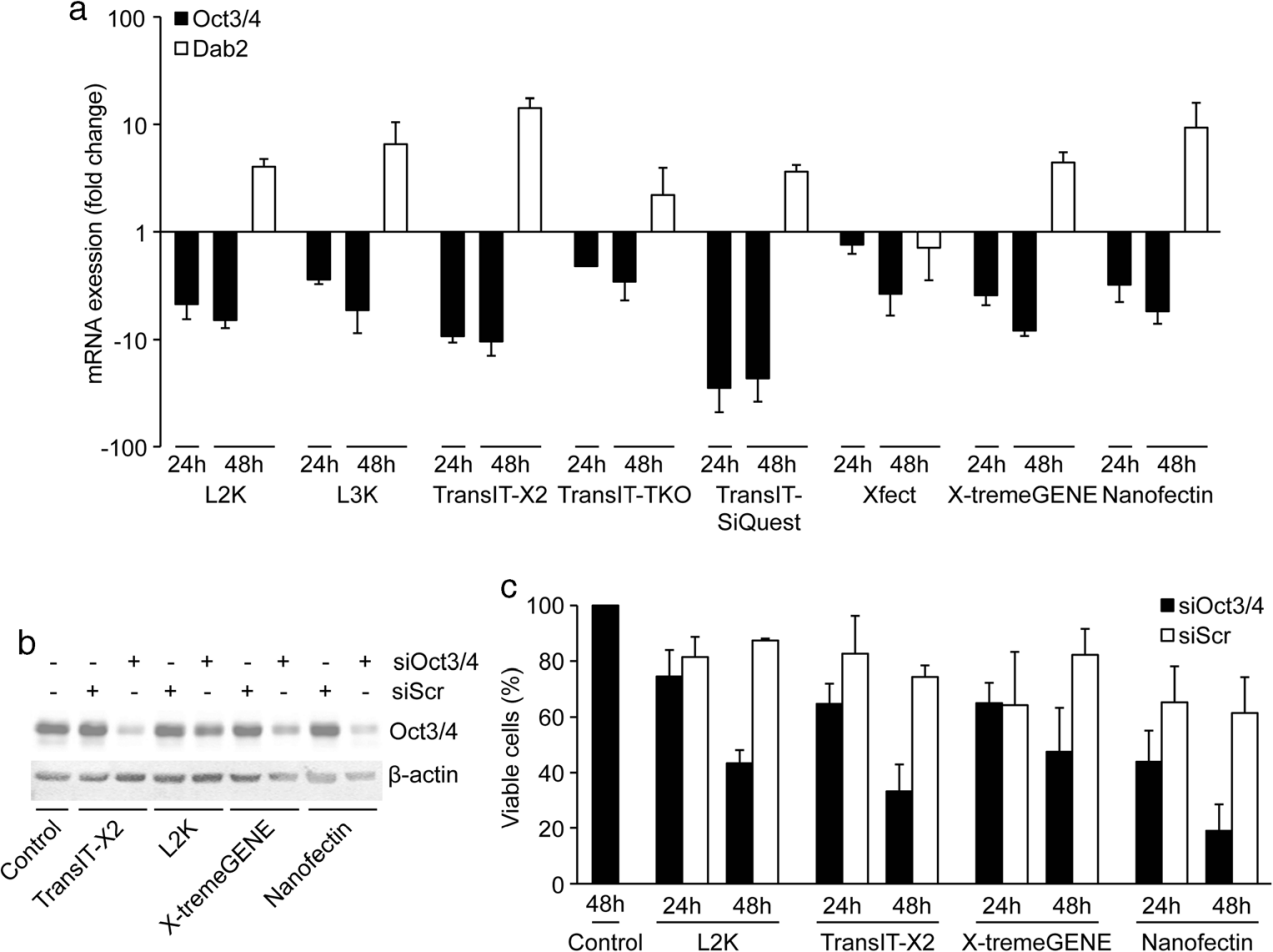
**a** Quantitative PCR analysis for Oct3/4 (24 and 48 h) and Dab2 (48 h) after transfection of E14 mES cells with either 100 nM siOct3/4 or scrambled siRNA using L2K (2 μl), L3K (1.5 μl), TransIT-X2® (3 μl), TransIT-TKO® (3 μl), TransIT-SiQuest® (5 μl), Xfect™ mESC (8 μl), X-tremeGENE™ siRNA (5 μl), and Nanofectin siRNA (8 μl). 18S expression was used for normalization, and results are comparative Ct value means ± SD (*n* = 3). **b** Western blot analysis for β-actin and Oct3/4 72 h post-transfection with siOct3/4 or scrambled siRNA using the four best-performing reagents in 1 a. **c** Trypan blue dye exclusion assay for cell viability 24 and 48 h post-transfection with siOct3/4 or scrambled siRNA using the same reagents as in 4b. Results are mean ± SD (*n* = 3)

The liposomal-based Lipofectamine2000 (L2K) and Lipofectamine 3000 (L3K), which allegedly works equally well for both siRNA and plasmid DNA, and the polymer-based TransIT-X2 TransIT-TKO, TransIT-siQUEST, X-tremeGENE siRNA, Xfect mESC and Nanofectin siRNA were evaluated. E14 mES cells were transfected, according to the manufacturers’ instructions, with 100 nM of siRNA against the pluripotency gene Oct3/4, or scrambled siRNA as negative control and subsequently analyzed for Oct3/4 expression by qPCR after 24 and 48 h. All transfection reagents significantly reduced Oct3/4 mRNA expression at both 24 and 48 h post-transfection, as compared to cells transfected with 100 nM scrambled siRNA (Fig. 1a). The most efficient Oct3/4 knockdown were achieved with TransIT-X2, TransIT-siQUEST and X-tremeGENE siRNA, showing more than 80 % decrease of Oct3/4 mRNA levels after 48 h. L2K, L3K and Nanofectin siRNA mediated 70 % decrease, whereas TransIT-TKO and Xfect mESC achieved around 50 % knockdown. Although, TransIT-siQUEST mediated the greatest reduction in Oct4 mRNA levels, the reagent generated more acute toxicity as compared to TransIT-X2 and X-tremeGENE and was therefore omitted from further analysis.

Oct3/4 down-regulation is known to lead to differentiation of mES cells [4]. Hence, to determine the efficacy of the siRNA mediated Oct3/4 knockdown, expression of the early endoderm differentiation gene Dab2 was assessed in the same qPCR samples as used for assessing Oct3/4 levels 48 h post-transfection (Fig. 1a). As expected, the samples with the best Oct3/4 knockdown showed the highest increase in Dab2 mRNA levels 48 h post-transfection. Western blotting for Oct3/4 protein expression after 72 h of transfection further confirmed the high knockdown efficacy obtained with the four most efficacious siRNA delivery reagents as assessed by level of RNA interference and cell survival (Fig. 1b). Trypan blue exclusion assay was then used to assess the toxicity of these four reagents 24 and 48 h post-transfection with the scrambled siRNA. The results showed no significant differences between the various reagents when using scrambled siRNA independent of time-point although Nanofectin appeared slightly more toxic to the cells (Fig. 1c). Cells transfected with Oct4 siRNA showed reduced viability as compared to cells transfected with scrambled siRNA and survival decreased over time suggesting that the Oct4 knockdown in combination with transfection negatively affects cells survival.

### Efficiency of DNA-Plasmid Delivery in Adherent Cells

**Fig. 2:**
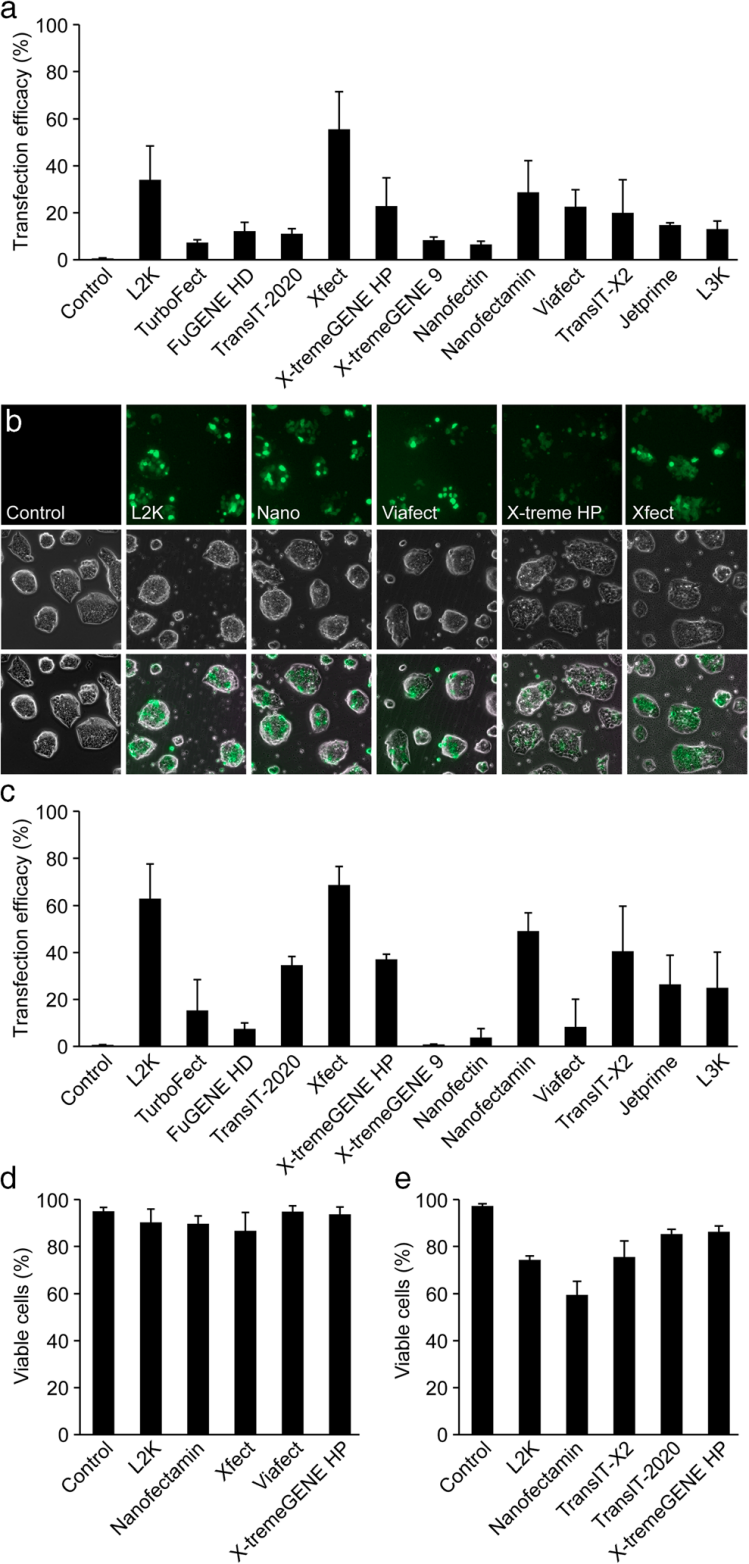
**a** Flow cytometry quantification of GFP-expressing E14 mES cells 24 h post-transfection of adherent cultures using L2K (DNA/reagent ratio 1:4), TurboFect™ (1:4), FuGENE® HD (1:4), TransIT-2020® (1:4), Xfect™ mESC (2:1), X-tremeGENE™ HP (1:1), X-tremeGENE™ 9 (1:6), Nanofectin (1:3), Nanofectamin (1:6), ViaFect™ (1:6), TransIT-X2® (1:2), JetPrime™ (1:2), and L3K (1:3) Results are mean ± SD (*n* = 2). **b** Representative fluorescence micrographs 24 h post-transfection with pMAX-GFP of adherent E14 mES cell cultures using the five best-performing reagents from 1 A. **c** Flow cytometry quantification of GFP-expressing E14 mES cells 24 h post-transfection in single cell suspension using L2K (DNA/reagent ratio 1:4), TurboFect™ (1:4), FuGENE® HD (1:4), TransIT-2020® (1:2), Xfect™ mESC (2:1), X-tremeGENE™ HP (1:1), X-tremeGENE™ 9 (1:6), Nanofectin (1:3), Nanofectamin (1:6), ViaFect™ (1:6), TransIT-X2® (1:2), JetPrime™ (1:2), and L3K (1:3) Results are mean ± SD (*n* = 2). **d-e** Trypan blue dye exclusion assay for cell viability 24 h post-transfection of adherent cultures (**d**) and single cell suspensions (**e**) using the five best-performing reagents from 1 A and 1C. Results are mean ± SD (*n* = 2)

Three liposomal based reagents (L2K, L3K, and Nanofectamin) as well as nine non-liposomal polymer based reagents (TurboFect, FuGENE HD, TransIT-2020, TransIT-X2, Xfect mESC, X-tremeGENE HP, X-tremeGENE 9, ViaFect, JetPrime) were evaluated for DNA-plasmid transfection. In addition, we also included Nanofectin; a positively charged polymer embedded into a porous carrier nanoparticle. The mES cell line E14 was transfected with pmaxGFP, an expression vector holding the gene for an advanced version of enhanced green fluorescent protein (GFP) under the control of the cytomegalovirus (CMV) promoter. In parallel experiments the cells were mock transfected to serve as negative controls. Twenty-four hours post-transfection cells were processed for flow cytometry analysis. The efficacy varied markedly between reagents (Fig. 2a). The highest efficiency was observed using the Xfect mESC transfection reagent, exhibiting 55 % GFP-positive cells when used at a DNA/reagent ratio of 2:1. The liposomal-based transfection reagents L2K (DNA/reagent ratio 1:4) and Nanofectamin (1:6) both exhibited efficiencies of 34 % and 29 %, respectively, while the remaining reagents never achieved more than 25 % transfection efficiencies. The five best-performing reagents were examined under fluorescent microscopy (Fig. 2b) and a trypan blue exclusion assay was performed 24 h post-transfection to evaluate toxicity for these reagents. All tested reagents maintained an 85 % survival rate or higher with no significant differences between groups (Fig. 2d).

### Efficiency of DNA-Plasmid Delivery in Single Cell Suspension

**Fig. 3:**
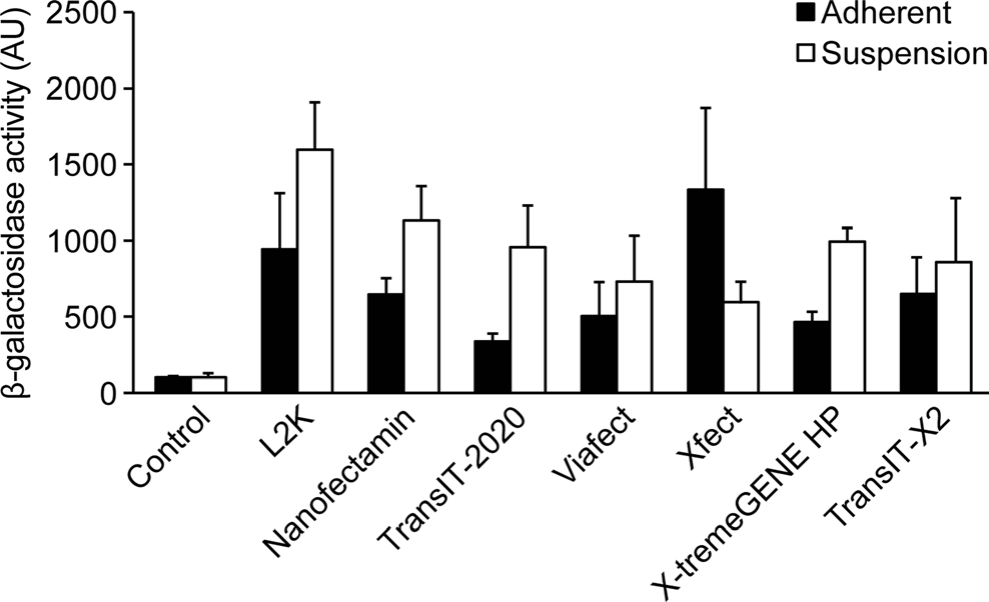
Beta-galactosidase activity assay 24 h post-transfection with pCMV β-galactosidase in E14 mES cells using L2K (DNA/reagent ratio 1:4), Nanofectamin (1:6), TransIT-2020® (1:2), ViaFect™ (1:6), Xfect™ mESC (2:1), X-tremeGENE™ HP (1:1), and TransIT-X2® (1:2). Results are mean ± sd (*n* = 2)

To assess whether 2i-cultured cells are more easily transfected when dissociated into single cells, E14 mES cells were trypsinized and then directly transfected using the same DNA/reagent ratios as presented above. For all transfection reagents 500.000 cells/sample were transfected with 1.5 μg pmaxGFP for two hours and subsequently seeded onto plates in 2i medium supplemented with 2 % FBS. Twenty-four hours post-transfection cells were processed for GFP expression using FACS analysis. Although Xfect generated the highest transfection efficiency (Fig. 2c), this reagent was found to be highly toxic to cells in suspension and only a small fraction of the cells survived and could re-attach to plates after transfection. This is also reflected by the lowest amount of β-gal activity compared to the other tested reagents (Fig. 3). In effect, there were too few cells to even perform the trypan blue exclusion assay. In contrast, the liposomal-based transfection reagents, L2K (1:4) and Nanofectamin (1:6) generated acceptable transfection rates of 63 % and 49 %, respectively with good survival (Fig. 2c). Viability was assessed 24 h post-transfection for the five most efficient reagents using trypan blue exclusion assay. Lower viability was observed when transfecting trypsinized cells as compared to adherent cultures and the toxicity level coincided with the transfection rate (Fig. 2e).

The five most efficient reagents from the initial screenings were then used for transfecting cells with a β-galactosidase (β-gal) reporter construct. The β-gal activity results concurred with the results presented above, both as to the efficiency of the different reagents but also that transfection rate of single cells exceeds that of adherent cultures (Fig. 3).

### Fast and Efficient DNA-Plasmid and siRNA Delivery in Suspended mES Cells

**Fig. 4:**
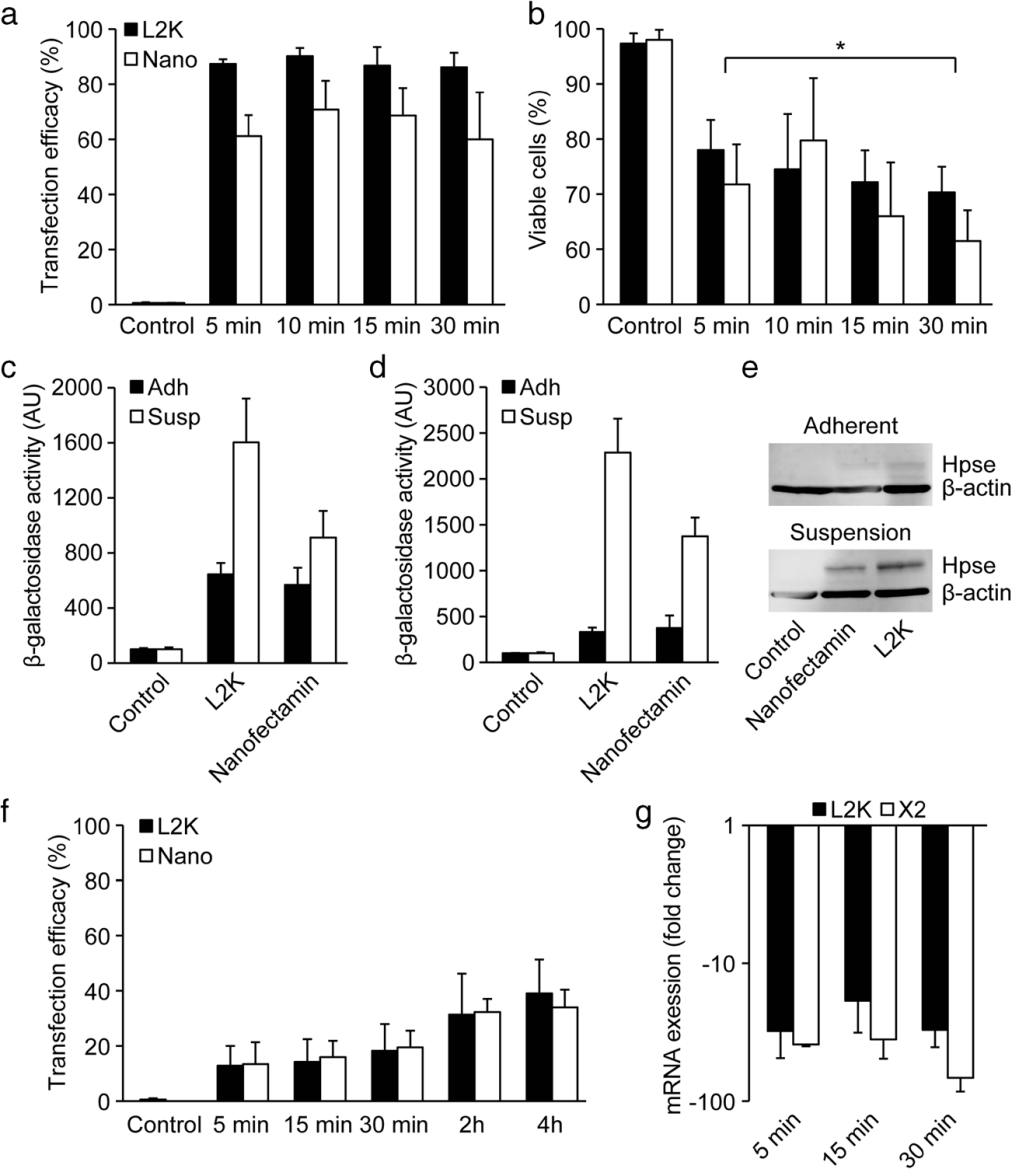
**a** Flow cytometry quantification of GFP-expressing E14 mES cells 24 h after short-time (5, 10, 15 and 30 min) transfection of single cell suspensions using L2K (DNA/reagent ratio 1:2) and Nanofectamin (1:6). Results are mean ± SD (*n* = 3) **b** Trypan blue dye exclusion assay for cell viability 24 h 24 h after short-time (5, 10, 15 and 30 min) transfection of single cell suspensions using L2K (DNA/reagent ratio 1:2) and Nanofectamin (1:6). Results are mean ± SD (*n* = 3). **c-d** Beta-galactosidase activity assay 24 h post-transfection with pCMV β-galactosidase using L2K (1:2) and Nanofectamin (1:6) of adherent cultures and single cell suspensions of E14 (**c**) and R1 (**d**) mES cells. **e** Western blot analysis for β-actin and heparanase 24 h post-transfection with pCAGGs-heparanase of adherent cultures or single cell suspensions of E14 mES cells. **f** Flow cytometry quantification of GFP-expressing E14 mES cells 24 h after short-time (5, 15 and 30 min) and standard-time (2 and 4 h) transfection of adherent cultures (DNA/reagent ratios 1:2). Results are mean ± SD (*n* = 3). **g** Quantitative PCR analysis for Oct3/4 24 h after short-time (5, 10, 15 and 30 min) transfection of single cell suspensions with 100 nM siOct3/4 or scrambled siRNA using L2K and. 18S expression was used for normalization, and results are comparative Ct value means ± SD (*n* = 2)

In an attempt to decrease toxicity and further optimize transfection of trypsinized mES cells DNA/reagent ratios were altered and shorter transfection times were tested. Since L2K had generated the most promising results we chose this reagent for the initial experiments. A similar set-up, as presented above, was used using the GFP reporter construct at DNA/reagent ratios of 1:1, 1:2 and 1:4. The 1:2 and 1:4 DNA/reagent ratios was found to be significantly more efficient than the 1:1 ratio (supplemental Fig. 1a). However, since no difference between the 1:2 and 1:4 ratios could be discerned, we continued with the 1:2 ratio to reduce toxicity. Transfection times were then decreased from two hours to 60 and 30 min. Our results showed that the transfection rates were similar independent of incubation time (supplemental Fig. 1b). The transfection time was subsequently further shortened to 15, 10 and 5 min. For these experiments we also included Nanofectamin (1:6). Again no significant differences in transfection efficiency were observed between the different incubation times (Fig. 4a). Hence, only 5-min incubation in suspension was needed to obtain about 90 % transfection efficacy with L2K and about 60 % with Nanofectamin. Furthermore, the toxicity seemed to decrease with shorter suspension times and was significantly lower with the 5-min transfection time (Fig. 4b). To corroborate our results we subsequently compared the 5-min single cell suspension transfection to cells cultured adherently in 2i media. Both L2K and Nanofectamin show significantly higher β-gal activity 24 h post-transfection after 5-min single cell suspension transfection compared to standard adherent cell transfection procedures in both E14 and R1 mES cells (Fig. 4c and 4d). In addition, E14 mES cells were transfected with a construct encoding the endoglycosidase heparanase. The apparent difference in expression levels of heparanase as shown by Western blotting further substantiates the advantages of the single-cell transfection procedure (Fig. 4e). Next, to see whether these short transfection conditions are applicable to adherent conditions as well, we shortened the transfection times for DNA-plasmid delivery in adherent cultures. However, our results showed that in adherent cultures there is a clear correlation between incubation time and transfection efficiency for both L2K and Nanofectamin (Fig. 4f).

Finally, to validate if short-time single cell suspension transfection works equally well for siRNA delivery, similar set-up, as presented above, was tested using L2K and TransIT-X2 with 100 nM Oct3/4 siRNA. Oct3/4 expression was markedly reduced 24 h post-transfection with 5, 15 and 30 min incubation (Fig. 4g). In fact, the level of Oct3/4 expression was clearly lower than after similar transfections in adherent 2i media cultures (Fig. 1a). Hence, as expected, these novel transfection conditions seem to work equally well for both plasmid-DNA as well as for siRNA delivery.

## Discussion

Over the years various biological, chemical, and physical strategies for introducing nucleic acids into cells have been developed. Today there are numerous commercially available products optimized for certain cell or nucleic acid types. Stem cells and primary cell cultures are often difficult to transfect. Consequently, working with these cells often means accepting low transfection efficiencies or laborious optimizations. Instead many studies are carried out in easily transfectable cell lines, such as HEK293 or HeLa, despite being poor cell models. Multilayered mES cell colonies give poor transfection efficiency due to the lack of homogenous cellular access for transfection reagents and nucleic acid complexes. As we have previously reported this quandary is even more troublesome when growing the cells in 2i media [2]. Moreover, post-transfection intrusion on cellular proteostasis (e.g. due to expression of plasmid DNA-encoded genes) and subsequent decrease in proliferation rates result in cultures being promptly overtaken by the less affected non-transfected cells. Although the latter is a common concern it becomes more critical for mES cells due to their excessive proliferation rates and alternate day sub-culturing needs.

In the present study we evaluated a panel of commonly used non-viral commercially available transfection reagents with regard to their applicability to deliver plasmid DNA and siRNA into mES cells cultured in 2i media. TransIT-X2 was found to be the most efficient reagent for siRNA transfection of adherent mouse ES cells cultured in 2i medium. When transfecting adherent cultures with DNA plasmid, the Xfect mESC non-liposomal polymer was the only reagent that generated more than 50 % transfection rate. However, L2K followed by Nanofectamin were the most efficient reagents when transfecting dissociated single cell suspensions, suggesting that lipofection is the method of choice for this experimental approach. Although Xfect mES reagents at first glance seemed equally efficient as L2K, the reagent was very toxic to cells in suspension. Suspension transfection is not mentioned in the manufacturer’s instructions for the Xfect mESC reagent. However, the traditional Xfect transfection reagent has been shown to work for suspension cell cultures [10 ].

It is likely that the inefficient transfection of cells cultured in 2i medium is partly due to the densely packed cell cultures making the transfection agent less prone to access all the cells. Enzymatic dissociation induces shaving of membrane-associated factors, including glycoproteins with highly sulfated glycosaminoglycan (GAGs) polysaccharide chains [11 ]. The large number of sulfate groups renders the chains anionic, which allows interaction with a wide variety of positively charged substrates. Previous studies have shown that cell surface GAGs interact with transfection reagent polymers and liposomes and inhibit cation-mediated gene transfer [12 ]. Hence, the increase in transfection efficiency and transgene expression observed after single cell suspension transfections is likely a result of a combination of the homogenous cellular accessibility for the transfection complexes in addition to a temporary removal of GAGs from the plasma membrane in response to trypsinization.

In an effort to fine-tune and reduce toxicity we optimized the use of L2K for plasmid-DNA delivery in single mES cell suspension. When optimizing transfection – whether for stem cells or other cell types – various cell-related factors such as culture health, passage numbers, and culture confluence need to be taken into consideration. However, those variables will be highly research group- and cell lab-dependent. Instead, we focused on parameters such as ratios of nucleic acid to transfection reagent and incubation time. We show that a DNA/reagent ratio of 1:2 rendered the best efficacy without significantly increasing toxicity. Also, by reducing incubation times to just 5 to 10 min we could maintain a high transfection efficacy but significantly increase cell viability. In fact, completely omitting the suspension incubation, and instead seeding the cells directly after adding the reagent and DNA-complexes, yielded only a slight decrease in transfection efficacy but a viability comparable to those obtained in adherent culture transfections (data not shown). Furthermore, similar results were obtained using R1 mES cells showing that the high efficiency and low toxicity is independent of cell line. Similar approaches have previously been reported for other cell types [13 – 15 ]. Although the focuses in those studies were on siRNA delivery, the efficiency for plasmid-DNA delivery was also tested. In concordance with our results these studies reported that a 10–15 min incubation of trypsinized cells with transfection complexes using L2K increases transfection efficiency 2–9 fold and that incubation times longer than that decrease cell viability. Consequently, this rapid transfection approach, which is both more time-efficient and gentle than the standard protocol, seems generally applicable to numerous cell types. In conclusion, the preferred method for plasmid DNA delivery of mESCs, cultured in 2i medium, is by transfecting the cells in suspension using L2K at a DNA:L2K ratio of 1:2 for 5–10 min. This gives a transfection rate of >80 % with a survival of >75 %. Similarly, the most efficient method for siRNA mediated knockdown is by transfecting cells with TransIT-X2 or L2K for 5–30 min, which mediates a > 90 % knockdown of Oct3/4. In addition, transfecting trypsinized cells effectively shortens the experimental time by one day as a result of not having to plate the cells 24 h prior to transfection as is required when transfecting adherent cells.

In the past, cationic lipid and polymer-based transfection had yet to reach the levels observed with viral transduction. With the presented method we have shown that this is no longer the case and that high-efficient transfections are achievable using non-viral methods. The optimized methods presented herein offers solutions to the current need for efficient and fast transfection in order to simplify and enhance studies of individual genes in pluripotent stem cells. Moreover, the method of transfecting trypsinized cells would be very advantageous in large scale and high-throughput transfection applications.

## Electronic Supplementary Material

Supplemental Figure 1 **A.** Flow cytometry quantification of GFP-expressing E14 mES cells 24 h post-transfection of single cell suspensions using L2 K (DNA/reagent ratios 1:1, 1:2 and 1:4). **B.** Flow cytometry quantification of GFP-expressing E14 mES cells 30 min, 1 h and 2 h post-transfection of single cell suspensions using L2 K (DNA/reagent ratio 1:2). Results are mean ± SD (*n* = 2) (GIF 15 kb)

High Resolution Image (TIFF 228 kb)

## References

[1] Ying QL, Wray J, Nichols J (2008). The ground state of embryonic stem cell self-renewal. Nature.

[2] Tamm C, Pijuan Galitó S, Annerén C (2013). A comparative study of protocols for mouse embryonic stem cell culturing. PloS One.

[3] Marks H, Kalkan T, Menafra R (2012). The transcriptional and epigenomic foundations of ground state pluripotency. Cell.

[4] Guo G, Pinello L, Han X (2016). Serum-based culture conditions provoke Gene expression variability in mouse embryonic stem cells as revealed by single-cell analysis. Cell Reports.

[5] Stavridis MP, Lunn JS, Collins BJ, Storey KG (2007). A discrete period of FGF-induced Erk1/2 signalling is required for vertebrate neural specification. Development.

[6] Wray J, Kalkan T, Gomez-Lopez S (2011). Inhibition of glycogen synthase kinase-3 alleviates Tcf3 repression of the pluripotency network and increases embryonic stem cell resistance to differentiation. Nature Cell Biology.

[7] Kim TK, Eberwine JH (2010). Mammalian cell transfection: the present and the future. Analytical and Bioanalytical Chemistry.

[8] Schaffer DV, Koerber JT, Lim KI (2008). Molecular engineering of viral gene delivery vehicles. Annual Review of Biomedical Engineering.

[9] Tamm C, Galito SP, Anneren C (2011). Differential effects on cell motility, embryonic stem cell self-renewal and senescence by diverse Src kinase family inhibitors. Experimental Cell Research.

[10] Arukuusk P, Parnaste L, Oskolkov N (2013). New generation of efficient peptide-based vectors, NickFects, for the delivery of nucleic acids. Biochimica et Biophysica Acta.

[11] Vogel KG (1978). Effects of hyaluronidase, trypsin, and EDTA on surface composition and topography during detachment of cells in culture. Experimental Cell Research.

[12] Ruponen M, Honkakoski P, Tammi M, Urtti A (2004). Cell-surface glycosaminoglycans inhibit cation-mediated gene transfer. The Journal of Gene Medicine.

[13] Zhang M, Guller S, Huang Y (2007). Method to enhance transfection efficiency of cell lines and placental fibroblasts. Placenta.

[14] Yang HY, Vonk LA, Licht R (2014). Cell type and transfection reagent-dependent effects on viability, cell content, cell cycle and inflammation of RNAi in human primary mesenchymal cells. European Journal of Pharmaceutical Sciences.

[15] Ma Y, Jin J, Dong C (2010). High-efficiency siRNA-based gene knockdown in human embryonic stem cells. RNA.

