# Supplementary figures and images for: Fast and Efficient Transfection of Mouse Embryonic Stem Cells Using Non-Viral Reagents
Source: Stem Cell Rev. 2016 Jun 30;12(5):584–91. doi: 10.1007/s12015-016-9673-5 (PMC5050252; doi:10.1007/s12015-016-9673-5)

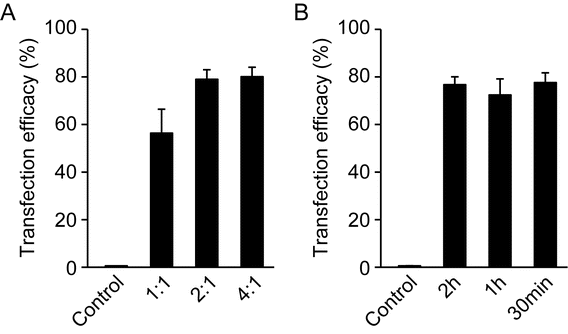

Supplement: Supplementary file 1 — A. Flow cytometry quantification of GFP-expressing E14 mES cells 24 h post-transfection of single cell suspensions using L2 K (DNA/reagent ratios 1:1, 1:2 and 1:4). B. Flow cytometry quantification of GFP-expressing E14 mES cells 30 min, 1 h and 2 h post-transfection of single cell suspensions using L2 K (DNA/reagent ratio 1:2). Results are mean ± SD (n = 2) (GIF 15 kb) [file 12015_2016_9673_Fig5_ESM.gif]

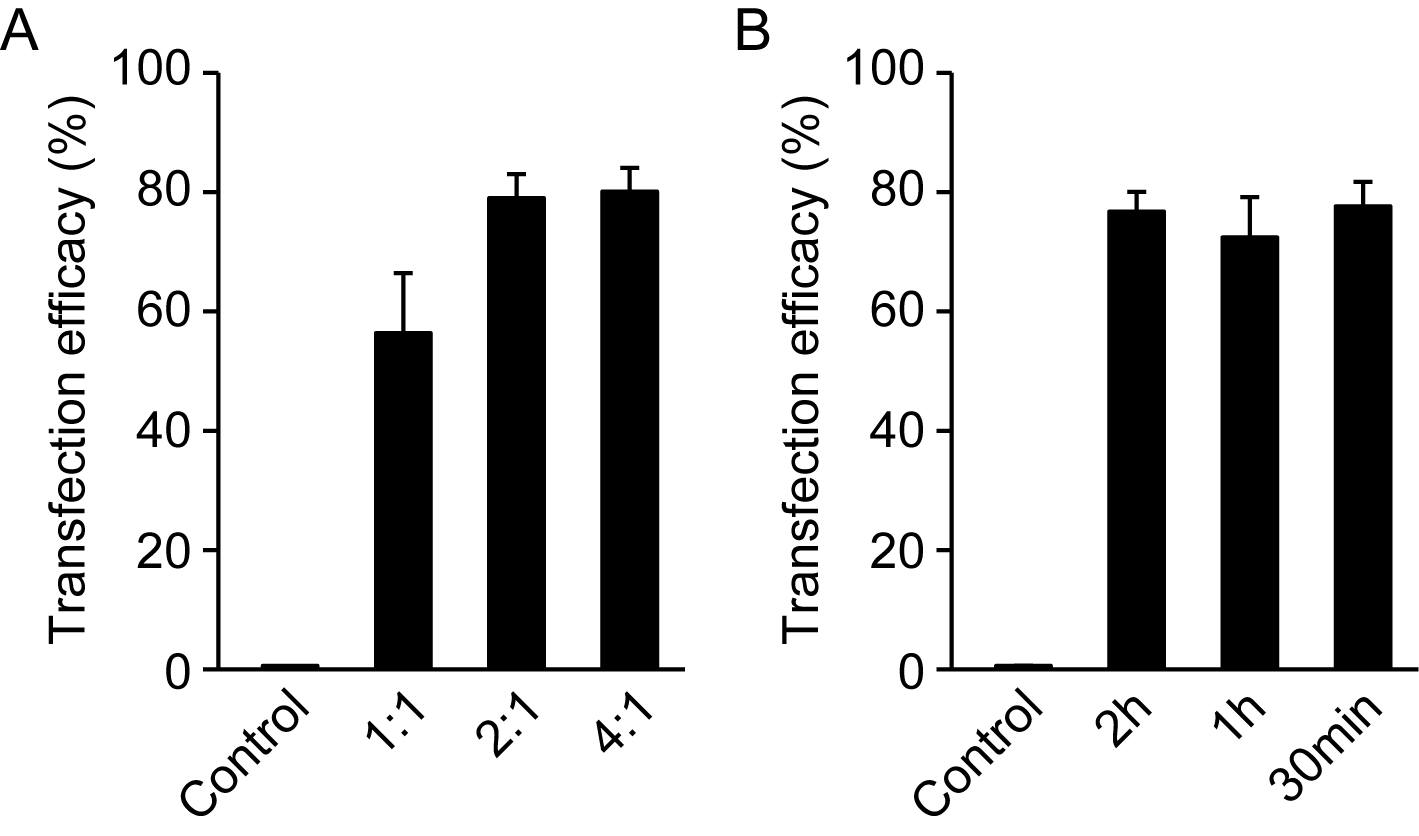

Supplement: Supplementary file 2 — High Resolution Image (TIFF 228 kb) [file 12015_2016_9673_MOESM1_ESM.tif]
